# Endurance Training Exercise Dose in Coronary Artery Disease Rehabilitation

**DOI:** 10.3390/jcdd12040134

**Published:** 2025-04-03

**Authors:** Daria Neyroud, Aaron L. Baggish

**Affiliations:** 1 Institute of Sport Sciences, Faculty of Biology and Medicine, University of Lausanne, 1015 Lausanne, Switzerland; daria.neyroud@unil.ch; 2 Department of Cardiology, University Hospital of the Canton de Vaud (CHUV), 1011 Lausanne, Switzerland

**Keywords:** exercise dose, cardiac rehabilitation, exercise frequency, exercise volume, exercise intensity, exercise duration, high-intensity interval training, coronary artery disease secondary prevention

## Abstract

Clinical management of patients with atherosclerotic coronary artery disease (CAD) following acute coronary syndrome includes cardiac rehabilitation. The well-established hallmark of cardiac rehabilitation is structured aerobic exercise training. To date, however, a limited number of studies have directly compared the effects of different doses of exercise on cardiovascular health, leaving uncertainty about the possible differential benefits of different exercise doses for use during cardiac rehabilitation. To address this area of uncertainty, we conducted a literature review and comparative analyses of studies that both compared two or more exercise interventions and assessed pre- and post-intervention peak oxygen consumption ($\dot{V}O_{2PEAK}$). Results from these analyses suggest that high exercise intensity, even when performed over relatively short duration interventions, appears to yield the most substantial improvements in cardiorespiratory fitness. However, this conclusion is based on the limited number of available studies, underscoring the need for future work examining exercise dose and clinical outcomes in the cardiac rehabilitation setting.

## 1. Introduction

Atherosclerotic coronary artery disease (CAD) is a leading cause of morbidity and mortality [1,2]. Clinical management of patients with CAD integrates percutaneous or surgical revascularization, pharmacotherapy, and lifestyle intervention [3,4]. Among patients presenting with an acute coronary syndrome (ACS), clinical guidelines recommend participation in cardiac rehabilitation following revascularization and the initiation of therapy to promote disease stabilization, with exercise training being the hallmark of cardiac rehabilitation [3,4].

Exercise training, not unlike pharmaceutical therapy, is defined by dose. Exercise dose is classically considered as the triple product of intensity, duration, and frequency [5,6]. Physical activity guidelines for primary prevention of cardiovascular disease recommend 150 min per week of moderate intensity [defined as activity requiring >4 metabolic equivalents (METs) of energy expenditure] exercise or 75 min per week of vigorous intensity (defined as activity requiring >6 METs of energy expenditure) exercise [7]. These dose recommendations stem from the broad-based epidemiologic literature which documents the impact of exercise doses on longevity.

Exercise dose used in cardiac rehabilitation is more variable both as a function of recommendations and clinical implementation. In general, clinical guidelines provide ranges rather than discrete targets of exercise duration, exercise intensity, and exercise frequency [3,4]. While this approach provides flexibility on a patient-by-patient basis and facilitates individualized dose tailoring based on patient characteristics, it promotes uncertainty about optimal dosing strategies. Historically, moderate intensity continuous exercise (MICT) protocols have represented the standard of care in cardiac rehabilitation. More recently, the efficacy and safety of alternative approaches, including high-intensity interval training (HIIT), have been demonstrated and applied in clinical practice. While the overall impact of both MICT and HIIT on clinical outcomes have been previously examined, we are unaware of prior work that has isolated the fundamental dose components of exercise in the rehabilitation setting with an emphasis on their impact on maximal oxygen consumption (i.e., the maximal volume of oxygen that an organism can consume, $\dot{V}O_{2MAX}$).

Accordingly, this paper was written to provide a critical appraisal of exercise dose data in the cardiac rehabilitation setting. Specifically, we compiled and examined studies that compared multiple doses of exercise with measurement of peak oxygen consumption ($\dot{V}O_{2PEAK}$) before and after the exercise intervention. We chose to closely examine and compare prior studies that compared multiple cardiac rehabilitation protocols in anticipation that these studies, of which there are few, would provide the most rigorous control of exercise dose. The fundamental goal of this review is to dissect different cardiac rehabilitation exercise protocols into their principal exercise dose principal components (i.e., intensity, duration, and frequency) to examine how each dose component individually relates to changes in $\dot{V}O_{2PEAK}$.

## 2. Exercise Prescription

Guidelines from leading clinical and scientific societies all recommend prescribing an exercise training regimen following an individualized frequency, intensity, time, type, volume, and progression model (FITT-VP). While exercise frequency (i.e., the number of exercise sessions performed in a given week), exercise time (i.e., the duration of a given exercise session), exercise type (i.e., exercise modality and/or pattern), and exercise volume (i.e., the total amount of exercise) are easily quantifiable, several methods exist to quantify exercise intensity. In cardiac rehabilitation settings, exercise intensity is most commonly described as a function of some maximal parameters such as a percentage of maximal heart rate, a percentage of maximal heart rate reserve, or a percentage of $\dot{V}O_{2MAX}$ [8]. In practice, with respect to aerobic training, current guidelines recommend an exercise dose corresponding to exercising 3 or more days per week at moderate to high intensity with each session lasting >20 min [9,10,11]. Unfortunately, the loose FITT-VP recommendations along with the absence of a standard measurement of exercise intensity impede our understanding of how manipulation of the principal dose parameters may maximize cardiac rehabilitation outcomes.

## 3. Lessons from the STRRIDE Study: The Importance of Exercise Dose

While not specifically focused on the cardiac rehabilitation setting, the landmark STRRIDE study provided important information about the importance of exercise dose. Initiated in 1998, the STRRIDE study investigated the effects of variable doses of aerobic exercise training on people (age range: 40–65 years) being overweight or obese (entry criteria body mass index = 25–37 kg/m^2^) and presenting with dyslipidemia [entry criteria of low density lipoprotein (LDL) = 130–190 mg/dL or high density lipoprotein (HDL) = <35 mg/dL for men and <45 mg/dL for women] [12]. Participants were randomized to one of four “exercise dose” groups: (i) *moderate-intensity* (40–55% of peak oxygen consumption, $\dot{V}O_{2PEAK}$)/*low-dose* (14 kcal/kg of energy expenditure per week) aerobic exercise, (ii) *high-intensity* (65–80% of $\dot{V}O_{2PEAK}$, i.e., corresponding to a heavy to severe exercise intensity)/*low-dose* (14 kcal/kg of energy expenditure per week) aerobic exercise, (iii) *high*-*intensity* (65–80% of $\dot{V}O_{2PEAK}$)/*high-dose* (23 kcal/kg of energy expenditure week) aerobic exercise, and (iv) non-exercise control group [12]. As participants were sedentary at baseline, a specified study entry criterion, they participated in several months of gradually increasing exercise intensity and duration to reach the dose to which they were randomized. Thereafter, the exercise interventions were performed for 6 months. For each participant, individualized weekly training duration was calculated to ensure adequate caloric expenditure. During the 6-month training intervention, participants in the *moderate-intensity/low-dose* group performed ~176 min of exercise over 3.4 sessions per week, participants in the *high-intensity/low-dose* group performed ~117 min of exercise over 3.0 sessions per week, and participants in the *high*-*intensity/high-dose* group performed ~174 min of exercise over 3.8 sessions per week.

Results from the STRRIDE study provide valuable insights into the importance of exercise dose with respect to discrete outcomes. First, all exercise regimens lead to an increase in $\dot{V}O_{2PEAK}$, but the *high-intensity/high-dose* program led to the greatest improvement [13,14]. Second, and similarly, all exercise regimens led to reductions in body mass [13,15,16,17], with the *high-intensity/high-dose* program resulting in the greatest body mass reduction [15]. Corollary reductions in visceral and subcutaneous fat were seen only in the *high-intensity/high-dose* program [15,17]. Third, blood lipid profiles were variably responsive to exercise in a dose-dependent fashion. Specifically, HDL increased only among participants assigned to the *high-intensity/high-dose* program [13], while the *moderate-intensity/low-dose* program appeared superior with regards to reduction in very-low-density lipoprotein [18]. Finally, fasting insulin was reduced and insulin sensitivity was improved following the *moderate-intensity/low-dose* and the *high-intensity/high-dose* programs but not following the *high-intensity/low-dose* program, thereby suggesting the primary importance of exercise duration for this outcome [16]. In summary, the STRRIDE experience demonstrates the importance of exercise dose with respect to discrete clinical characteristics and thereby suggests that tailoring dose based on desired outcomes is required for optimal results to be obtained in clinical settings. 

## 4. Exercise Dose Response for the Secondary Prevention of CAD

While results from the STRRIDE study clearly highlight the importance of identifying the optimal exercise dose for a given clinical endpoint, two primary impediments currently preclude the standardization of exercise dose for cardiac rehabilitation. First, there is a relative dearth of data that rigorously examine fundamental components of exercise dose in relation to desired clinical outcomes such as change in $\dot{V}O_{2MAX}$ or $\dot{V}O_{2PEAK}$. Second, there are relatively few studies that have directly compared different exercise dose regimens in a cardiac rehabilitation setting using carefully controlled study designs.

In an attempt to clarify the current state of knowledge regarding exercise dose in cardiac rehabilitation, we conducted a broad-based literature review and a comparative analysis of studies that met the following two criteria. First, studies selected for inclusion were required to have compared two or more exercise interventions with different exercise doses and to have reported the fundamental components of exercise dose (session frequency, duration, and intensity) for each intervention studied. Second, studies selected for inclusion were required to provide pre- and post-intervention measurements of $\dot{V}O_{2PEAK}$. Studies meeting these criteria are summarized in Table 1 [19,20,21,22,23,24,25,26,27,28]. Using this framework, we examined the relationships between exercise dose and key determinants of health in cardiac rehabilitation for the secondary prevention of CAD.

Across the included studies (n = 10), participants were predominantly middle-aged men undergoing exercise training interventions with durations ranging from 4 to 14 weeks. In all studies, exercise dose was controlled by alteration of exercise intensity and duration and/or pattern (i.e., continuous vs. intervals), while training frequency was kept constant throughout each intervention. Of note, only two studies included a non-exercise control group. Six studies compared moderate-intensity continuous training (MICT) to high-intensity interval training (HIIT) [20,21,22,24,25,28], two studies compared HIIT to maximal-intensity interval training (MIIT) [23,27], one study compared continuous low-intensity (LICT) to continuous high-intensity (HICT) training [19], and one study compared HIIT to an aerobics class [26]. Several studies manipulated the total exercise dose of a given exercise session [19,21,27], while others manipulated specific exercise dose parameters while keeping the overall exercise dose constant [24,25,28]. In aggregate, data from these studies provide information about how different outcomes may be affected by different exercise doses or, for a given dose, by different exercise intensities and/or patterns.

### 4.1. Exercise Dose and Cardiorespiratory Fitness

$\dot{V}O_{2MAX}$ is the gold standard measure of cardiorespiratory fitness and is determined by the integrated function of the respiratory, cardiovascular, and muscular systems. Low cardiorespiratory fitness is an independent risk factor for cardiovascular disease and all-cause mortality [29,30,31,32]. Two large studies conducted in cardiac rehabilitation patients showed that an increase of 1 mL.kg^−1^.min^−1^ translates to a 9–10% improvement in prognosis in both men and women [31,32]. $\dot{V}O_{2MAX}$, therefore, represents an important clinical endpoint and is often used to determine the efficacy of clinical rehabilitation. Note that maximality of the cardiopulmonary exercise test is not always achieved and only rarely reported in method sections (Supplementary Table S1). As such, here we will use the $\dot{V}O_{2PEAK}$ terminology to discuss changes in the maximal $\dot{V}O_{2}$ measured during the cardiopulmonary exercise test.

**Figure 1:**
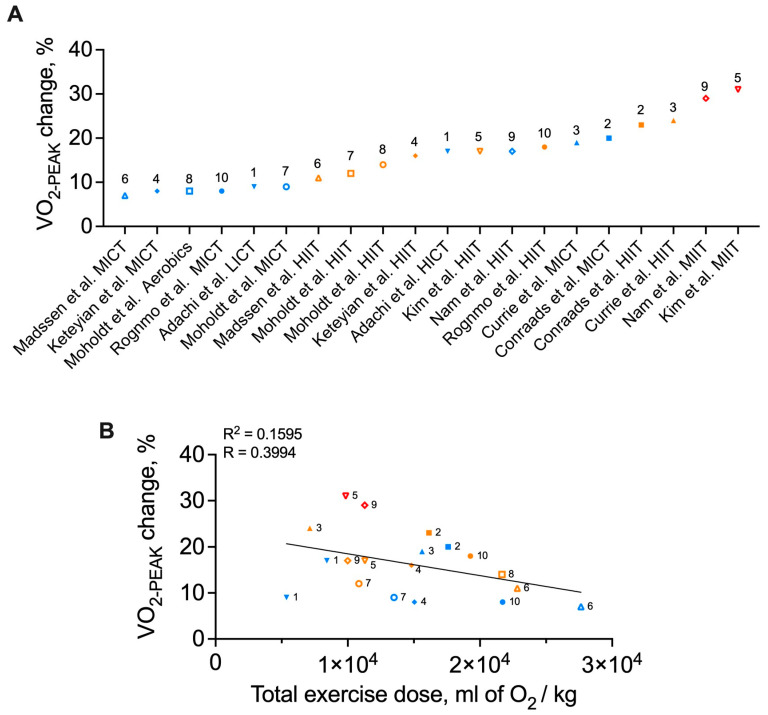
Changes in $\dot{V}O_{2PEAK}$ following cardiac rehabilitation exercise training regimens in patients with coronary artery disease following an acute coronary syndrome or revascularization (**A**) and the relationship between the $\dot{V}O_{2PEAK}$ response and total exercise dose (**B**). Numbers indicated above each data point correspond to Table 1 study number [19,20,21,22,23,24,25,26,27,28]. Each study is represented by a given symbol. Exercise interventions consisting of continuous paradigms are depicted in blue, of high-intensity intervals in orange, and of maximal-intensity intervals in red. Exercise dose reflects the estimated total amount of oxygen consumed during the entire training intervention calculated as the product of exercise intensity (converted in percentage of $\dot{V}O_{2PEAK}$ when needed), exercise session duration, exercise frequency, and intervention duration. HIIT = high-intensity interval training; HICT = high-intensity training; LICT = low-intensity continuous training; MICT = moderate-intensity continuous training; MIIT = maximal-intensity interval training.

Table 1 and Figure 1 summarize the changes in $\dot{V}O_{2PEAK}$ elicited by the different exercise training regimens employed in the studies identified by our review of the literature [19,20,21,22,23,24,25,26,27,28]. Six of these studies compared MICT to HIIT [20,21,22,24,25,28], and all found either similar increases in $\dot{V}O_{2PEAK}$ across both interventions [20,21,25] or a greater increase in VO_2PEAK_ with HIIT [22,24,28]. Currie et al. demonstrated similar increases in $\dot{V}O_{2PEAK}$ with HIIT (+24%) and MICT (+19%), but it is noteworthy that HIIT sessions from this study were designed to elicit a lower overall training load compared to the MICT sessions. This suggests that HIIT performed at comparably lower total exercise dose can lead to similar increase in $\dot{V}O_{2PEAK}$ compared to MICT [21]. In contrast, Moholdt et al. reported similar increases in $\dot{V}O_{2PEAK}$ in response to isoenergetic MICT (+9%) and HIIT (+12%) [25]. Compared to Currie et al., their HIIT intervention utilized longer time intervals (4 × 4 min vs. 10 × 1 min), higher training frequency (5 days/week vs. 3 days/week), but an overall shorter training intervention (4 weeks vs. 12 weeks), suggesting a role for flexibility in the design of HIIT interventions. Despite these inconsistencies in the above summarized results, higher intensities of training appear to be associated with greater increases in $\dot{V}O_{2PEAK}$. Further support for this observation emerges from studies that compared differential intensity levels but kept exercise duration and frequency constant [19,23,27]. However, it must be acknowledged that increasing exercise intensity while maintaining exercise duration and frequency resulted in an increase in total exercise dose. Accordingly, it is possible that increases in exercise duration and frequency with maintenance of lower intensities would translate into similar increases in $\dot{V}O_{2PEAK}$. Future studies with multiple regimen comparisons will be required to resolve this uncertainty.

**Figure 2:**
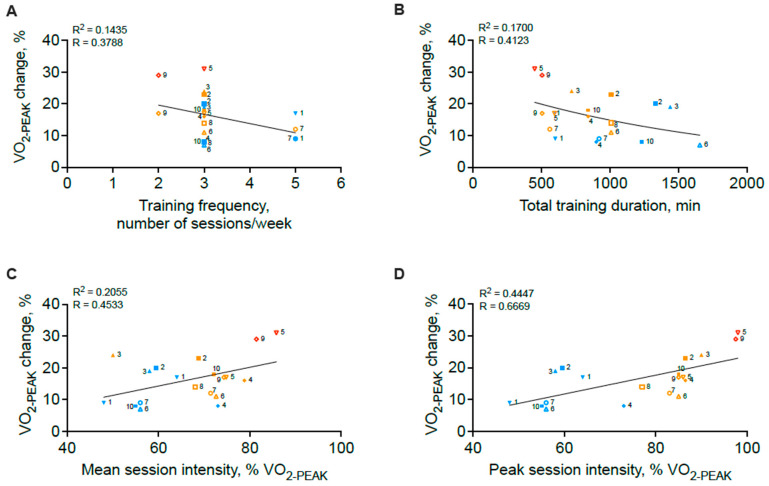
Relationships between the $\dot{V}O_{2PEAK}$ response and exercise frequency (**A**), exercise duration (**B**), mean exercise session intensity (**C**), and peak exercise session intensity (**D**). Numbers indicated with each data point correspond to Table 1 study number. Each study is represented by a given symbol. Exercise interventions consisting of continuous paradigms are depicted in blue, of high-intensity intervals in orange, and of maximal-intensity intervals in red.

In an effort to further delineate how total exercise dose and its fundamental components (i.e., frequency, intensity, and duration) impact changes in VO_2PEAK_, we extracted and compared changes in $\dot{V}O_{2PEAK}$ (Δ$\dot{V}O_{2PEAK}$) data from each study (Figure 1A). We observed a range of $\dot{V}O_{2PEAK}$ increases across studies ranging from 7 to 31%. We next examined relationships between total exercise dose, the fundamental components of exercise dose, and the $\dot{V}O_{2PEAK}$ response. For these analyses, total exercise intervention dose was calculated as the total oxygen consumption over the entire training intervention, assuming that all participants fully adhered to the prescribed training protocol. We further assumed that all participants maintained their daily-life physical activities levels outside of structured training so that the exercise training intervention represented the only training stimulus. Exercise intensity was standardized by converting reported exercise intensity to a percentage of measured $\dot{V}O_{2PEAK}$ for each training session. Exercise frequency was defined as the number of training sessions per week. Finally, exercise duration represents the total number of minutes of training during the entire intervention. Descriptions of “warm-up” and “cool-down” protocols were often vague and thus were excluded from our calculations. The relationships between total exercise dose, intensity, frequency, duration, and change in $\dot{V}O_{2PEAK}$ are shown in Figure 1B and Figure 2.

**Figure 3:**
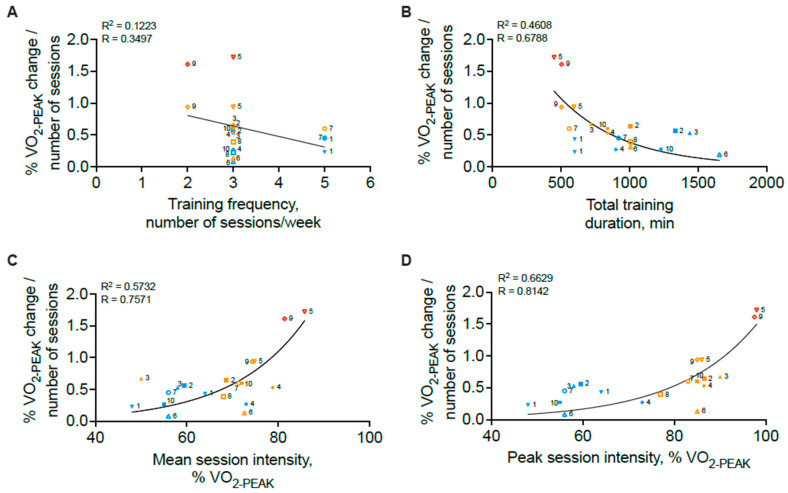
Relationships between the VO_2PEAK_ response adjusted for total number of exercise sessions in each training regimen and exercise frequency (**A**), exercise duration (**B**), mean exercise session intensity (**C**), and peak exercise session intensity (**D**). Numbers indicated with each data point correspond to Table 1 study number. Each study is represented by a given symbol. Exercise interventions consisting of continuous paradigms are depicted in blue, of high-intensity intervals in orange, and of maximal-intensity intervals in red.

The first noteworthy observation is the strong indirect trend between total exercise intervention dose and $\dot{V}O_{2PEAK}$ response (Figure 1B). As exercise dose is a composite reflection of exercise intensity, duration, and frequency, the observed $\dot{V}O_{2PEAK}$ response was next examined as a function of each parameter. Training frequency and total training duration were indirectly associated with the $\dot{V}O_{2PEAK}$ response (Figure 2A,B). In contrast, mean session intensity and peak session intensity were directly related to the $\dot{V}O_{2PEAK}$ response and explained approximately 21% and 44% of the $\dot{V}O_{2PEAK}$ response, respectively (Figure 2C,D). To account for the potential impact of the total duration of exercise intervention, which differed widely across interventions, we next adjusted the $\dot{V}O_{2PEAK}$ response by the total number of exercise sessions performed in each exercise intervention (Figure 3). Here again, we note indirect relationships between training frequency, total training duration, and the $\dot{V}O_{2PEAK}$ response (Figure 3A,B) but strong direct curvilinear relationships between mean and peak session intensity and the $\dot{V}O_{2PEAK}$ response (Figure 3C,D). It should be noted that the negative relations observed between $\dot{V}O_{2PEAK}$ improvements and total exercise dose (Figure 1B), training frequency (Figure 2A and Figure 3A), and total training duration (Figure 2B and Figure 3B) were largely driven by the HIIT/MIIT interventions which tended to have the lowest frequency and duration values but the highest intensity values. In aggregate, these analyses suggest that high exercise intensity, even when performed over relative short duration interventions, yield the most substantial improvements in cardiorespiratory fitness. This finding is relevant, as high- and medium-intensity interval training protocols are increasingly popular in cardiac rehabilitation settings and have been associated with acceptable safety profiles [33,34]. In addition, our data demonstrate a “trade-off” effect in which higher-intensity exercise protocols typically utilize shorter durations of training performed in intervals. For instance, in all the studies reviewed here, all exercise protocols eliciting a mean or peak session intensity greater than 65% of $\dot{V}O_{2MAX}$ utilized an interval exercise paradigm (Figure 2C,D). Future studies, aiming to include both high intensity coupled with higher frequency and/or total duration, will be required to determine if these combinations may yield substantially better improvements in VO_2PEAK_.

### 4.2. Beyond $\dot{V}O_{2MAX}$: Cardiac Risk Factors and Quality of Life

While cardiorespiratory fitness represents a key outcome for secondary prevention exercise interventions, other parameters associated with cardiovascular health deserve consideration. For instance, obesity is a well-known risk factor for cardiovascular disease. Among the 10 studies summarized above, only four examined how different exercise interventions impacted body mass, and each reported no significant changes in body mass in response to MICT, aerobics, HIIT, or MIIT [23,25,26,28]. Blood pressure, a key determinant of cardiovascular disease progression [35], was only assessed in four studies before and after the training [19,20,21,22], with data from these studies indicating either no changes [19,22] or similar reductions in response to LICT and HICT or MICT and HIIT [20,21]. Of note, one study reported a trend toward diastolic blood pressure reduction (*p* = 0.051) following HICT but not LICT [19], while similar reductions in diastolic blood pressure were observed in response to the HIIT and MICT interventions performed by Currie et al. [21]. Four studies assessed the impact of different exercise training regimens on vascular health and showed similar increases in flow-mediated dilation (i.e., an index of endothelial function) in response to 12 weeks of MICT or aerobics and HIIT [20,21,24,26]. In addition, 12 weeks of MICT or HIIT led to similar changes in coronary plaque structure [24]. In aggregate, the limited information available to date precludes definitive determinations about the impact of exercise dose on key determinants of cardiovascular health in the rehabilitation setting.

Parameters of metabolism and inflammation represent additional clinical endpoints of secondary prevention interventions. Five of ten studies in Table 1 examined blood lipid levels before and after their exercise interventions with variable results. Short-duration interventions of MICT or HIIT, performed 5 days per week over 4 weeks, lead to no significant changes in HDL, LDL, or triglyceride concentrations [25]. The reported impact of longer-duration exercise training on plasma lipids is inconsistent. Specifically, 12 weeks (three sessions per week) of either MICT or HIIT led to an increase in HDL [20], and 12 weeks of HIIT (three sessions per week), but not aerobics training, led to an increase in HDL [26]. In contrast, Madssen et al. reported no changes in total cholesterol, HDL, or LDL in response to 12 weeks of MICT or HIIT performed 3 days per week [24]. Lastly, one study examining 6 weeks of MICT and HIIT performed 3 days per week reported reductions in LDL with both interventions [23]. In aggregate, these results suggest that training interventions lasting longer than 4 weeks are required to induce changes in HDL or LDL, with the exercise intensity or exercise pattern possibly being important for HDL but not LDL. Future work to explain the heterogeneity of the lipid response to exercise training is warranted.

Three of these aforementioned studies also measured the glucose response to training, and two measured plasma adiponectin and ferritin concentrations [24,25,26]. No changes in glucose concentrations were observed following any of the training interventions tested (i.e., aerobics, MICT, or HIIT), possibly suggesting that higher exercise doses might be required. Levels of ferritin were found to be similarly reduced following 4 weeks of MICT (−28%) or HIIT (−28%), or following 12 weeks of aerobics (−19%) or HIIT (−7%), suggesting that independent of exercise intensity, relatively short training interventions lead to reductions in ferritin concentrations. Finally, adiponectin, a hormone involved in glucose metabolism, fatty acid catabolism, and inflammation, was unchanged after 4 weeks of MICT or HIIT [25] but increased after 12 weeks of either aerobics or HIIT [26], suggesting that training interventions longer than 4 weeks might be necessary to induce changes in adiponectin.

Quality of life was evaluated in five of the studies analyzed in this review [20,24,25,26,27]. These studies reported similar quality of life improvement when comparing HIIT to MICT or HIIT to aerobics. Nam et al. reported that quality of life improved in non-exercised patients during the early recovery phase of an acute MI while patients who performed either MICT or HIIT showed greater quality of life improvements than those performing no exercise [27]. Taken together, these findings support the ability of secondary prevention exercise training interventions to improve quality of life, but the limited available data preclude definitive conclusions about whether a specific exercise dose, intensity, duration, or frequency might be preferential.

## 5. Methodological Limitations to Consider

In addition to the dearth of studies that rigorously examined how different exercise durations, frequencies, and intensities affect clinical outcomes, several methodological limitations further confound our understanding of the exercise dose–response relationship in cardiac rehabilitation. First, and as already mentioned above, exercise protocols are often incompletely described, lacking information about details regarding warm-ups, cool-downs, or participant adherence. Second, prescribing exercise intensity as a percentage of maximal effort (be it heart rate, heart rate reserve, or $\dot{V}O_{2MAX}$) relies on (i) patients being able to exert maximally and (ii) the assumption that a given percentage of maximal effort results in the same metabolic equivalents across different patients (see Hansen et al. 2019 [36] for a detailed discussion of that topic). Patients commonly terminate cardiopulmonary exercise testing at submaximal levels due to symptom occurrence. Even in cases where true maximal effort is achieved, one cannot assume that a given percentage of any maximal metric will result in the same physiological and metabolic demand [36]. For example, 75% of $\dot{V}O_{2PEAK}$/$\dot{V}O_{2MAX}$ may lie within the heavy exercise intensity domain (i.e., defined as exercised intensity lying between the first and second ventilatory thresholds) for a given patient and within the severe intensity domain (i.e., above the second ventilatory threshold) for another patient. Such differences in the relative intensities at which patients actually exercise prevent rigorous assessment of the relationship between exercise intensity and cardiac rehabilitation-induced improvements in fitness [37].

## 6. Future Directions

For exercise to be used as medicine with optimal efficacy and individualization, a more detailed understanding of its dose–response relationship is needed. As highlighted by the present work, our current understanding of how different exercise doses and patterns differently contribute to reductions in specific cardiac risk factors and improved cardiorespiratory fitness is limited. Future clinical and scientific advances will require (i) a change in our approach to the study of exercise, (ii) detailed reporting of individual responses as a function of adherence to the exercise prescription, and (iii) rigorous reporting of exercise dose parameters in publications. Indeed, if exercise is to be considered as medicine, it should be studied in a manner analogous to that used for the development of pharmaceutical compounds. The study of pharmaceutical compounds follows a well-defined scripted sequence, requiring an initial assessment of the dose–response relationship (Phase 1), followed by the study of the biological mechanisms of action (Phase 2) and human subject variability (Phase 3). In contrast, the majority of prior work examining the effects of exercise have largely focused on small-cohort responses to one arbitrary exercise dose, with only relatively few studies included in this review having examined two or more exercise doses in the same clinical setting. As evidenced by the data synthesis provided in this review, comparison of exercise dose across different studies is complicated by the variability in how exercise dose, especially exercise intensity, is reported (e.g., percentage of maximal heart rate, percentage of heart rate reserve, percentage of $\dot{V}O_{2PEAK}$/$\dot{V}O_{2MAX}$, percentage of peak power output, etc.) and by incompleteness in the reporting of exercise dose parameters. Future research should aim to rigorously examine the exercise dose–response of given cardiovascular outcomes by careful manipulation and isolation of exercise intensity, exercise duration, exercise frequency, and exercise patterns. Finally, the focus of this review was confined to aerobic exercise dose. The importance of resistance training, often performed in parallel with aerobic training, is acknowledged, and a similar dissection of fundamental resistance training dose components (power, repetitions, sets, frequency, etc.) in cardiac rehabilitation represents a logical area of future work.

## 7. Conclusions

In conclusion, this review was designed to highlight current understandings of how exercise dose in the rehabilitation setting impacts cardiorespiratory fitness and other markers of cardiovascular risk. At present, there are insufficient data to arrive at a definitive conclusion about what levels of exercise intensity, duration, and frequency lead to optimal rehabilitation outcomes. While our analyses, which align with several recent meta-analyses conducted in both CAD and heart failure patients [38,39,40,41,42], suggest that intensity may be the most important of these three fundamental parameters of exercise dose, future work will be required to confirm this speculation.

## Figures and Tables

**Table 1:** Exercise training interventions evaluating the effect(s) of different exercise doses/patterns in cardiac rehabilitation.

(Study #)/Author/Reference	Inclusion Criteria	Exercise Intervention	Study Population	Frequency and Duration	Key Findings	Training Effect/Dose Effect
**(1)** Adachi et al. 1996 [19]	Post-MI	Walking *UC:* no structured physical activity *LICT:* 15 min/session at 80% of VT1 *HICT:* 15 min/session at HR of 40% ∆ $\dot{V}O_{2PEAK}$ HR—VT1 HR	Duration between MI and training onset = 48 ± 8 days n = 29 *UC:* 62 ± 9 y, 75% male *LICT:*63 ± 7 y, 91% male *HICT:*51 ± 11 y, 90% male	2x/day 5x/wk 2 mo	∆$\dot{V}O_{2PEAK}$*UC* = ↑ 10% (*p* = 0.09) *LICT* = ↑ 9% (*p* = 0.20) ***HICT* = ↑ 14% (*p* = 0.03)** Additional Findings: Similar ↓ in resting HR (average ~8 bpm) in all groups after training No ∆ in DBP or SBP (trend for ↓ DPB with HICT, *p* = 0.051)	Yes/yes Training effect only with *HICT*
**(2)** Conraads et al. 2015 [20]	Post-MI, post-PCI, or post-CABG	Cycling *MICT:* 47 min/session including 5 min warm-up, 37 min at 70–75% HR_PEAK_, 5 min cool-down*HIIT:* 38 min/session including 10 min of warm-up, 4 × 4 min at 90–95% HR_PEAK_ interspersed by 3 min at 50–70% HR_PEAK_, 3 min cool-down	Duration between acute event and training onset = 4–12 wk n = 200 *MICT:* 59.9 ± 9.2 y, 89% male *HIIT:* 57.0 ± 8.8 y, 91% male	3x/wk 12 wk	**∆** $\dot{V}O_{2PEAK}$ ***MICT* = 20.3% (*p* < 0.001)** ***HIIT* = 22.7% (*p* < 0.001)** Additional Findings: Similar ↑ in HR_PEAK_, O_2_ pulse, FMD, QoL, HDL-c, and total cholesterol in both groups Similar ↓ in resting DBP (and trend for SBP) and hs-CRP in both groups	Yes/no
**(3)** Currie et al. 2013 [21]	Post-MI, post-PCI, or post-CABG	Cycling *MICT:* 30–50 min/session at 58% of PPO *HIIT:* 20 min/session consisting of 10 × 1 min at 80–104% of PPO interspersed with 1 min at 10% of PPO HIIT = ½ training load of MICT	Patients referred to CR n = 22 *MICT:* 68 ± 8 y, 91% male *HIIT:* 62 ± 11 y, 91% male	3x/wk 12 wk	**∆** $\dot{V}O_{2PEAK}$ ***MICT* = 19% (*p* ≤ 0.001)** ***HIIT* = 24% (*p* ≤ 0.001)** Additional Findings: Similar ↑ in VO_2_ at VT2 and in FMD in both groups Similar ↓ in resting DPB and HR in both groups	Yes/yes
**(4)** Keteyian et al. 2014 [22]	Post-MI, post-PCI, or post-CABG; EF > 40%	Treadmill *MICT:* 40 min/session, including 5 min warm-up, 30 min at 60–80% HRR, 5 min cool-down*HIIT:* 40 min/session consisting of 5 min warm-up, 3 min at 60–70% HRR, 4 × 4 min at 80–90% HRR interspersed by 3 min at 60–70% HRR, 4 min cool-down	Patients enrolled in CR Duration between MI or PCI and training onset >3 wk Duration between CABG and training onset >4 wk n = 28 *MICT:* 58 ± 9 y, 92% male *HIIT:* 60 ± 7 y, 73% male	3x/wk 10 wk	**∆** $\dot{V}O_{2PEAK}$ ***MICT* = 8% (*p* ≤ 0.05)** ***HIIT* = 16% (*p* ≤ 0.05)** Additional Findings: Greater ↑ in VO_2_ at VT2 with HIIT No ∆ in DBP or SBP	Yes/yes
**(5)** Kim & Choi 2020 [23]	Post-ACS	Walking *HIIT:* 50 min/session consisting of 10 min warm-up at 50–70% HRR, 3 × 8 min at 85% HRR interspersed by 3 min at 40% HRR, 10 min cool-down at 50–70% HRR *MIIT:* 45 min/session including 10 min warm-up at 50–70% HRR, 4 × 4 min at 95–100% HRR interspersed by 3 min at 60% HRR, 10 min cool-down at 50–70% HRR	Duration between ACS and training onset > 3 wk n = 47 *HIIT:* 62.8 ± 11.9 y, 67% male *MIIT:* 60.0 ± 11.0 y, 78% male	3x/wk 6 wk	**∆** $\dot{V}O_{2PEAK}$ ***HIIT* = 17% (*p* < 0.05)** ***MIIT* = 31% (*p* < 0.05)** Additional Findings: Similar ↑ in HR_PEAK_ between groups (*p* = 0.052 for HIIT and 0.048 for MIIT), Similar ↓ in resting HR and LDL-c between groups No ∆ in HDL-c, triglycerides, LVEF, LVEDD, LVESV, and body mass	Yes/yes
**(6)** Madssen et al. 2014 [24]	Post-angina pectoris or non-ST elevation ACS following stent implantation	Walking/running *MICT:* 46 min/session at 70% HR_PEAK_ *HIIT:* 41 min/session consisting of 10 min warm-up, 4 × 4 min at 85–95% HR_PEAK_ interspersed with 3 min at 70% HR_PEAK_ MICT and HIIT = isocaloric	n = 36 *MICT:* 60.5 (56.5–63.5) y, 71% male *HIIT:* 55.5 (50.0–60.5) y, 93% male	3x/wk 12 wk	**∆** $\dot{V}O_{2PEAK}$ ***MICT* = 7% (*p* < 0.05)** ***HIIT* = 11% (*p* < 0.05)** Additional Findings: Similar ↑ in QoL (and trend for FMD, *p* = 0.07) between groups Similar changes in coronary artery plaque structure between groups No changes in BMI, total cholesterol, HDL, LDL, triglycerides, or glucose	Yes/yes
**(7)** Moholdt et al. 2009 [25]	Post-CABG	Treadmill walking *MICT:* 46 min/session at 70% HR_PEAK_ *HIIT:* 41 min/session consisting of 8 min warm-up, 4 × 4 min at 90% HR_PEAK_ interspersed with 3 min at 70% HR_PEAK_, 5 min cool-downHIIT and MICT = isocaloric	Duration between CABG and training onset = 4–16 wks n = 59 *MICT:* 62.0 ± 7.6 y, 77% male *HIIT:* 60.2 ± 6.9 y, 86% male	5x/wk 4 wk	**∆** $\dot{V}O_{2PEAK}$ ***MICT* = 9% (*p* < 0.001)** ***HIIT* = 12% (*p* < 0.001)** Additional Findings: Similar ↑ in HR recovery and QoL between groups Similar ↓ in ferritin levels between groups No changes in HDL, LDL, triglycerides, adiponectin, or glucose	Yes/no
**(8)** Moholdt et al. 2012 [26]	Post-MI	*Aerobic:* 60 min consisting of 10 min warm-up, 35 min of aerobic exercise (walking, jogging, lunges, squats) following music, 10 min cool-down*HIIT:*38 min of treadmill consisting of 8 min warm-up, 4 × 4 min at 85% HR_PEAK_ interspersed with 3 min at 70% HR_PEAK_, 5 min cool-down	Duration between MI and training onset = 2–12 wk n = 107 *Aerobics:* 57.7 ± 9.3 y, 83% male *HIIT:* 56.7 ± 10.4 y, 83% male	3x/wk 12 wk	**∆** $\dot{V}O_{2PEAK}$ ***Aerobics* = 7.5% (*p* < 0.001)** ***HIIT* = 14% (*p* < 0.001)** Additional Findings: ↓ in resting HR with Aerobics only ↑ in HDL with HIIT only Similar ↑ in FMD, adiponectin and QoL between groups Similar ↓ in ferritin between groups	Yes/yes
**(9)** Nam et al. 2024 [27]	Post-MI	Treadmill *UC:*instructed to exercise at an RPE of 11–13 with no restrictions placed on exercise activities*HIIIT:* 50 min consisting of 10 min at 40% $\dot{V}O_{2PEAK}$, 4 × 4 min at 85% $\dot{V}O_{2PEAK}$ interspersed with 3 min at 60% $\dot{V}O_{2PEAK}$, 10 min cool-down at 40% $\dot{V}O_{2PEAK}$*MIIT:* 50 min consisting of 10 min at 40% $\dot{V}O_{2PEAK}$, 4 × 4 min at 95–100% $\dot{V}O_{2PEAK}$ interspersed with 3 min at 60% $\dot{V}O_{2PEAK}$, 10 min cool-down at 40% $\dot{V}O_{2PEAK}$	Duration between MI and training onset = 1–2 wk n = 106 *UC:* 56.7 ± 9.5 y, 88% male *HIIT:* 58.7 ± 12.4 y, 86% male *MIIT:* 56.1 ± 10.5 y, 90% male	2x/wk 9 wk	**∆** $\dot{V}O_{2PEAK}$ ***UC* = 5% (*p* < 0.05)** ***HIIT* = 17% (*p* < 0.05)** ***MIIT* = 30% (*p* < 0.05)** Additional Findings: Greater ↑ in QoL with HIIT and MIIT vs. UC Greater ↑ in 6 MWT with MIIT vs. UC (no ∆ between MIIT and HIIT or HIIT and UC)	Yes/yes
**(10)** Rognmo et al. 2004 [28]	Post-MI, post-PCI, or post-CABG	Uphill treadmill walking *MICT:* 41 min at 50–60% $\dot{V}O_{2PEAK}$*HIIT:* 33 min consisting of 5 min warm-up at 50–60% $\dot{V}O_{2PEAK}$, 4 × 4 min at 80–90% VO_2PEAK_ interspersed by 3 min at 50–60% $\dot{V}O_{2PEAK}$, 3 min cool-down at 50–60% $\dot{V}O_{2PEAK}$ MICT and HIIT = same workload	Duration between MI and training onset >3 mo; duration between PCI/CABG and training onset > 12 mo n = 21 *MICT:* 61.2 ± 7.3 y, 89% male *HIIT:* 62.9 ± 11.2 y, 75% male	3x/wk 10 wk	**∆** $\dot{V}O_{2PEAK}$ ***MICT* = 7.9% (*p* < 0.05)** ***HIIT* = 17.9% (*p* < 0.05)** Additional Findings: No ∆ in DBP, SBP or body mass	Yes/yes

ACS = acute coronary syndrome; BMI = body mass index; bpm = beats per minute; CABG = coronary artery bypass grafting; CR = cardiac rehabilitation; DBP = diastolic blood pressure; EF = ejection fraction; FMD = flow-mediated dilation; HICT = high-intensity continuous training; HIIT = high-intensity interval training; HR = heart rate; HRR = heart rate reserve; LICT = low-intensity continuous training; MI = myocardial infarction; MICT = moderate-intensity continuous training; MIIT = maximal-intensity interval training; mo = month; PCI = percutaneous coronary intervention; PPO = peak power output; QoL = quality of life; SBP = systolic blood pressure; UC = usual care; $\dot{V}O_{2PEAK}$ = peak oxygen consumption recorded during the cardiopulmonary exercise test (i.e., note that given the methodologies provided by the majority of the studies included in this table, we cannot assume that $\dot{V}O_{2PEAK}$. corresponds to the maximal amount of oxygen that patients could consume; see Supplementary Table S1); VT1 = ventilatory threshold 1; VT2 = ventilatory threshold 2; wk = week; 6MWT = 6 min walk test; y = years; ↑ = increase; ↓ = decrease.

## Data Availability

No new data were created or analyzed in this study. Data sharing is not applicable to this article.

## Supplementary Materials

The following supporting information can be downloaded at: https://www.mdpi.com/article/10.3390/jcdd12040134/s1, Table S1: Cardiopulmonary exercise testing protocol used in exercise training interventions included in Table 1.
