# Supplementary material for: Endurance Training Exercise Dose in Coronary Artery Disease Rehabilitation
Source: J Cardiovasc Dev Dis. 2025 Apr 3;12(4):134. doi: 10.3390/jcdd12040134 (PMC12028330; doi:10.3390/jcdd12040134)
Supplement: Supplementary file 1 [file jcdd-12-00134-s001.zip › jcdd-3485816-supplementary.pdf]

Supplementary Table S1. Cardiopulmonary exercise testing protocol used in exercise training interventions included in Table 1.

(Study #)/ Author/ Reference	Cardiopulmonary exercise testing protocol	Cardiopulmonary exercise testing modality	Reason for terminating the cardiopulmonary exercise testing
**(1)** Adachi et al. 1996[19]	• 3 min warm-up at 20 W and 60 rpm<br>• 1 W increments every 6 s	Bike	• Symptom-limited termination
**(2)** Conraads et al. 2015[20]	• 10 or 20 W<br>• 10 or 20 W/min increments	Bike	• Not specified
**(3)** Currie et al. 2013[21]	• 1 min at 100 kpm and 70 rpm<br>• 100 kpm/min increments	Bike	• Exhaustion
**(4)** Keteyian et al. 2014[22]	• Modified-Bruce protocol	Treadmill	• Symptom-limited termination<br>• Participants encouraged to reach an RPE > 17
**(5)** Kim & Choi 2020[23]	• Modified-Bruce protocol	Treadmill	• RPE > 17<br>• RER > 1.1
**(6)** Madssen et al. 2014[24]	• Individually-adjusted test	Treadmill	• Not specified
(7) Moholdt et al. 2009[25]	• Ramp protocol individually adjusted to last 8-12 min	Treadmill	• Not specified
**(8)** Moholdt et al. 2012[26]	• Ramp protocol individually adjusted to last 8-12 min	Treadmill	• Not specified
**(9)** Nam et al. 2024[27]	• Modified-Bruce protocol	Treadmill	• RPE > 17 or<br>• RER > 1.1
**(10)** Rognmo et al. 2004[28]	• 10-min warm-up at confortable speed and slope<br>• 2% incline increments every 2 min	Treadmill	• Not specified

Kpm = kilopond (a gravitational metric of force), RER = respiratory exchange ratio, RPE = rate of perceived exertion.
